# Prior Virus Exposure Alters the Long-Term Landscape of Viral Replication during Feline Lentiviral Infection

**DOI:** 10.3390/v3101891

**Published:** 2011-10-13

**Authors:** Xin Zheng, Scott Carver, Ryan M. Troyer, Julie A. Terwee, Sue VandeWoude

**Affiliations:** Department of Microbiology, Immunology and Pathology, 1619 Campus Delivery, Colorado State University, Fort Collins, CO 80523, USA; E-Mails: xin.zheng@colostate.edu (X.Z.); scott.carver@colostate.edu (S.C.); ryan.troyer@colostate.edu (R.M.T.); julie.terwee@colostate.edu (J.A.T.)

**Keywords:** FIV, HIV, co-infection, lentivirus, feline, tropism, reservoir, immunodeficiency, interferon-gamma, innate immunity

## Abstract

We developed a feline model of lentiviral cross-species transmission using a puma lentivirus (PLV or FIV_Pco_) which infects domestic cats but does not cause disease. Infection with PLV protects cats from CD4+ T-cell decline caused by subsequent infection with virulent feline immunodeficiency virus (FIV). Previous studies implicate innate immune and/or cellular restriction mechanisms for FIV disease attenuation in PLV-infected cats. In this study, we evaluated viral infection and cytokine mRNA transcription in 12 different tissue reservoirs approximately five months post infection. We quantitated tissue proviral load, viral mRNA load and relative transcription of IL-10, IL-12p40 and IFNγ from tissues of cats exposed to FIV, PLV or both viruses and analyzed these parameters using a multivariate statistical approach. The distribution and intensity of FIV infection and IFNγ transcription differed between single and co-infected cats, characterized by higher FIV proviral loads and IFNγ expression in co-infected cat tissues. Variability in FIV mRNA load and IFNγ was significantly more constrained in co-infected *versus* singly infected cat tissues. Single-infected:co-infected ratios of FIV mRNA load compared to FIV proviral load indicated that active viral transcription was apparently inhibited during co-infection. These results indicate that previous PLV infection increases activation of tissue innate immunity and constrains the ability of FIV to productively infect tissue reservoirs of infection for months, independent of FIV proviral load, supporting a model in which innate immunity and/or modulation of target cell susceptibility play a key role in PLV-induced protection from FIV disease.

## 1. Introduction

Feline immunodeficiency virus (FIV) is a lentivirus closely related to human immunodeficiency virus (HIV) and simian immunodeficiency virus (SIV) that naturally infects numerous wild and domestic feline species. Individual feline species typically harbor genetically-distinct species-specific FIV strains [1–3]. Infection with the domestic cat (*Felis catus*) strain of FIV (FIV_Fca_) results in CD4+ T-cell depletion and pathogenic disease which progresses to AIDS-like immune dysfunction and ultimately death [4,5]. FIV infection and disease in domestic cats bears many similarities to HIV infection and AIDS in humans including similar routes of infection, cell and tissue tropism, clinical symptoms and course of disease [6,7]. Thus, FIV infection of the domestic cat is a useful animal model for studying HIV infection and vaccine development [4,6,8,9]. In contrast, FIV infection of nondomestic felid species has little measurable impact on survival of the natural host [2,10–13], but may be associated with long-term immune cell depletion and other disease sequelae [14–17]. Cross-species transmission events are thought to be limited by lack of contact between host species and the action of host-specific cellular restriction factors [18]. Experimental infection of domestic cats with a lentivirus (puma lentivirus (PLV) or FIV_Pco_) native to the cougar (*Puma concolor*) results in productive yet avirulent infection with no detectable T-cell depletion or clinical disease [19,20], providing a model to evaluate mechanisms for restriction of lentiviral cross-species infection. PLV viral load diminishes over time to virtually undetectable levels in circulation and lymphoid tissues with low level infection of the gastrointestinal tract [19]. Intensive sequence analysis of PLV genomes integrated in cat blood cells showed a strong bias toward G-to-A hypermutation [21], a hallmark of cellular cytidine deaminase-mediated viral restriction [22–25], suggesting that cellular restriction likely plays a role in control of PLV infection in domestic cats. Thus PLV infection is a model for cross-species transmission of a virus which is not well-adapted to long-term replication in the new host and remains nonpathogenic.

Previously we have studied whether infection of domestic cats with PLV can impart resistance to subsequent infection with virulent FIV [26,27]. Cats infected with PLV 28 days prior to FIV challenge were protected from the marked CD4+ T-cell depletion experienced by cats challenged with FIV alone [26]. PLV/FIV co-infected cats had a unique immunologic profile distinct from FIV single infected cats—including elevated levels of CD8, CD25 and FAS expressing cells and elevated expression of the cytokines IL-4 and IFNγ [26,28]. In contrast, we did not detect evidence of adaptive immunity to FIV such as neutralizing antibodies or virus-specific cytotoxic T-cells [26]. These data suggest that innate rather than adaptive immune mechanisms are associated with PLV-induced protection from FIV disease. Additionally, examination of FIV population genetics demonstrated that during PLV/FIV co-infection FIV underwent a population bottleneck at approximately three weeks post-infection not observed in FIV single infection [29]. The nature of this bottleneck remains to be determined, but the data are consistent with PLV-induced host restriction of normally virulent FIV. Collectively, these studies suggest that host innate immunity has a role in mediating PLV-induced modulation of FIV infection and disease. However, the exact nature of PLV-induced protection remains to be elucidated.

Our previous studies aimed at determining the mechanism(s) of PLV-induced protection from FIV disease have focused largely on timepoints early after FIV infection (<120 days post-PLV infection) using peripheral blood mononuclear cells (PBMC) for analysis [26,28,29]. In this study we focused on PLV and FIV infection and innate immune response in multiple infected cat tissues at a later chronic infection timepoint (159 days post-PLV infection) for the purpose of detecting changes in infection and immunity which may be specific to important tissue reservoirs of infection and provide insight into PLV mediated protection. Further, evaluating distribution of both provirus and viral RNA transcription across a suite of tissues allows analysis of the hypothesis that PLV infection alters the distribution and tissue reservoirs of susbsequent FIV infection.

We found that PLV co-infection altered the magnitude and variability of FIV infection and innate immunity in tissues compared to FIV single infection—to a remarkable extent considering our analyses were conducted nearly five months following co-infection. While somewhat surprisingly, FIV proviral load tended to be higher in tissues from co-infected *versus* single-infected animals, viral transcripts tended to be lower in the co-infection state. Comparison of FIV proviral load and FIV mRNA load among single-infected *versus* co-infected tissues indicated that PLV co-infection limits FIV productive infection (viral mRNA expression) relative to FIV proviral load. Collectively, these data suggest that PLV-induced protection from FIV disease may be at least partially mediated by persistent alterations of innate immunity resulting in limitation of FIV productive infection. It is also possible that restriction of target cell populations via PLV-induced immune activation or alteration of susceptibility for other reasons results in an altered FIV infection landscape. This hypothesis is supported by the finding that viral and cytokine transcription rates were more variable during single FIV infection, and as reported previously, FIV replication in the face of previous PLV infection is highly constrained during acute infection. If during co-infection, FIV is restricted to a cell type with longer half-life that is less permissive for viral replication, we would predict an outcome similar to that observed in this study. These experiments pose a new paradigm for assessment of protective immunity against HIV/AIDS—namely that perturbation of early innate immune parameters and circulating cell phenotype can alter the outcome of a virulent lentiviral infection.

## 2. Results

### 2.1. Multivariate Analysis of PLV/FIV Co-Infection Parameters in Infected Organs

**Figure 1.**
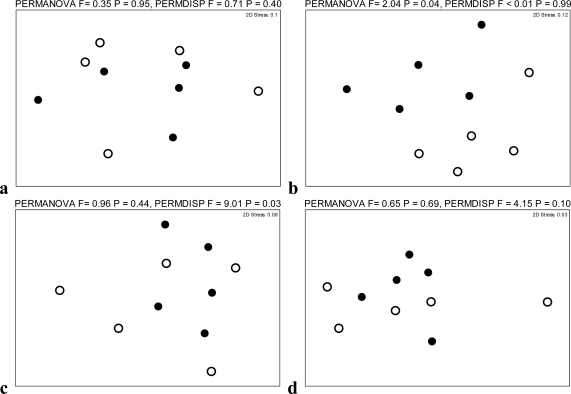

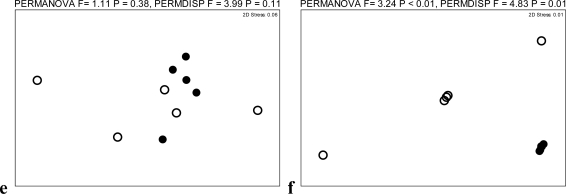
Non-metric multidimensional scaling (NMDS) ordination plots of proviral load, viral mRNA and cytokine mRNA among tissues for single virus infected (open circles) and co-infected (closed circles) cats: (**a**) Puma lentivirus (PLV) provirus, (**b**) Feline immunodeficiency virus (FIV) provirus, (**c**) FIV mRNA, (**d**) IL-10, (**e**) IL-12, and (**f**) IFNγ. Permutational based multivariate analysis of variance (PERMANOVA) examines the overall distribution and amount of provirus, viral mRNA and cytokine mRNA among tissues. Permutational analysis of multivariate dispersion (PERMDISP) evaluates if provirus, mRNA and cytokine loads of co-infected cats were more constrained among tissues than cats with single virus infections.

We sought to gain further insight into PLV-induced protection from FIV disease during the chronic phase of infection by characterizing the viral distribution and innate immune response within different anatomic compartments during PLV and FIV single and dual infections. For each of 12 organs (bone marrow, thymus, spleen, liver, pre-scapular lymph node (LN), mesenteric LN, Peyer’s patch, duodenum, jejunum, ileum, colonic LN, and tonsils) we determined the following parameters: PLV proviral load (an indicator of residual PLV infection), FIV proviral load (an indicator of residual FIV infection), FIV mRNA load (an indicator of productive FIV infection), and mRNA expression of the cytokines IL-10, IL-12p40 and IFNγ. To reduce the number of potential analyses resulting from this experiment we used a permutational based multivariate analysis of variance (PERMANOVA) to examine if there were differences between single and co-infected cats in the data matrix among tissues for each of the parameters above. This test permitted us to evaluate if the overall distribution and amount of provirus, viral mRNA and cytokines differed between single and co-infection. These data are graphically represented in Figure 1 using non-metric multidimensional scaling (NMDS) plots. Of the six parameters investigated by PERMANOVA, the distribution of FIV provirus and IFNγ significantly differed between FIV single and PLV/FIV co-infected cats (Figure 1). Thus, for these parameters, differences between single and co-infected cats for each individual tissue were examined further using generalized linear models (GLM, Sections 2.3 and 2.4 below). Results for parameters which did not significantly differ between FIV single and PLV/FIV co-infection (PLV provirus, FIV mRNA, IL-10 and IL-12) are available in Supplementary Data.

### 2.2. Multivariate Analysis of Data Dispersion for Single *versus* Co-Infection

In addition to PERMANOVA, we examined if variation in the distribution and amount of provirus, mRNA and cytokines of co-infected cats were more constrained (similar) among tissues, than cats with single virus infections. This analysis was undertaken using a permutational analysis of multivariate dispersion (PERMDISP). This test permitted us to evaluate if provirus, mRNA and cytokines of cats with co-infections were more similar to one another than the same measurement in cats with single virus infections. For FIV mRNA and IFNγ mRNA expression the differences among FIV/PLV co-infected cats were significantly smaller, or more constrained, than the differences among FIV single infected cats (Figure 1). This suggests that PLV co-infection generally constrains productive FIV infection as indicated by limited variability in FIV mRNA expression across the 12 tissues studied. Similarly, PLV co-infection appears to focus the innate immune response to FIV infection by limiting variability in IFNγ expression across tissues.

### 2.3. IFNγ mRNA Expression in Individual Tissues

**Figure 2.**
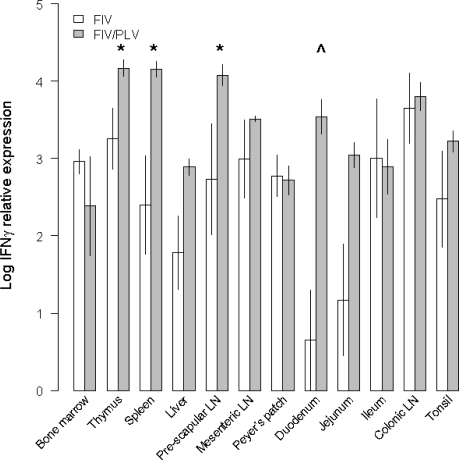
IFNγ expression (Log IFNγ mRNA expression relative to Glyceraldehyde-3-phosphate dehydrogenase (GAPDH) expression ± SE) among tissues for FIV single and FIV/PLV co-infected cats. Significant generalized linear model (GLM) comparisons (p < 0.05) are indicated by * and trends (p < 0.10) by ^ above bars (see Table 1).

GLM analyses were performed to further investigate the differences in IFNγ mRNA expression between FIV single and PLV/FIV co-infection at an individual tissue level. In previous studies we have found higher IFNγ expression in blood cells of co-infected cats compared to single infected cats [26,28], but this is the first study to examine IFNγ expression in infected tissues. IFNγ expression was significantly higher in FIV/PLV co-infected cats than FIV single infected cats in thymus, spleen and pre-scapular LN and a trend (p < 0.1) was observed in duodenum (Figure 2 and Table 1). Thus multiple infected tissues (typically those with higher FIV viral loads) showed a substantially altered innate immune response to FIV attributable to previous PLV infection.

### 2.4. FIV Provirus Distribution in Individual Tissues

**Figure 3.**
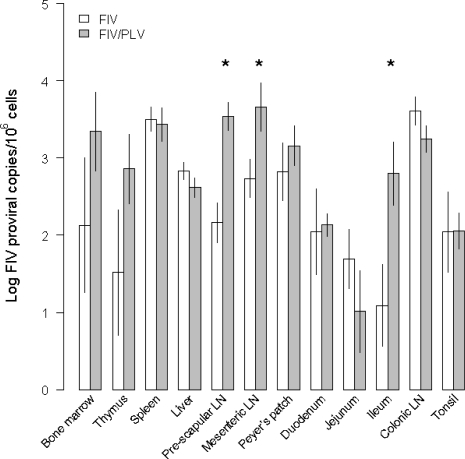
FIV proviral load (mean ± SE) among tissues for FIV single and PLV/FIV co-infected cats. Significant GLM comparisons are indicated by * above bars (see Table 1).

FIV proviral load was significantly greater in PLV/FIV co-infected cats than in FIV single infected cats in the pre-scapular lymph node (LN), mesenteric LN and ileum while in the other nine tissues no significant difference was detected (Figure 3 and Table 1). This result suggests that previous PLV infection does not generally limit the ability of FIV to infect important solid tissue targets of infection. In fact, previous PLV infection appeared to increase the ability of FIV to colonize the pre-scapular LN, mesenteric LN and ileum.

### 2.5. Inhibition of Productive FIV Infection by PLV Coinfection

With the surprising finding that previous PLV infection does not limit, and in fact may enhance, the ability of FIV to persist in cat tissues during subacute infection, we next sought to examine whether there was a difference in productive FIV infection in tissues of single and co-infected cats. While presence of FIV provirus indicates ability of the virus to infect cells, this value does not measure whether the integrated provirus is actively producing viral transcripts, an essential step in perpetuating viral infection and inducing immunodyscrasias. Intracellular FIV mRNA abundance was therefore measured as a better indicator of active viral transcription. While we did not find a statistically significant difference in total FIV mRNA between single and co-infection, FIV mRNA abundance trended higher for single infection compared to co-infection in 11 of 12 tissues examined (Supplementary Table 1) in contrast to the generally higher abundance of FIV provirus in co-infected cat tissues (Table 1).

**Figure 4.**
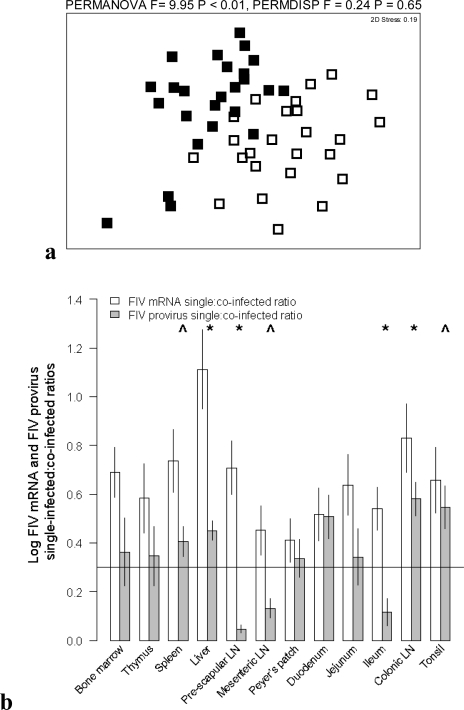
Inhibition of infection success (FIV provirus single:co-infected ratio) and productive FIV infection (FIV mRNA single:co-infected ratio) in co-infected cats among tissues. (**a**) NMDS ordination plot of single:co-infected ratios. (**b**) Single:co-infected ratios in individual tissues (mean ± SE). Above line indicates greater provirus or mRNA in single than co-infection tissues (greater inhibition in co-infected cats). Below line indicates greater provirus or mRNA in co-infected than single infected tissues (promotion in co-infected cats). Significant GLM comparisons (p < 0.05) are indicated by * and trends (p < 0.10) by ^ above bars (see Table 2).

In order to determine if productive infection (FIV mRNA expression) was inhibited in co-infected cats, we compared the single-infected:co-infected ratio of FIV mRNA to the single-infected:co-infected ratio of FIV provirus. To make this comparison, all possible individual pair-wise combinations of single-infected:co-infected ratios were first calculated for mRNA and provirus (n = 25 in each case). FIV mRNA single-infected:co-infected ratios and FIV provirus single-infected:co-infected ratios were then compared using PERMANOVA, PERMDISP and GLM procedures as described above. When the mRNA single-infected:co-infected ratio is less than the provirus single-infected:co-infected ratio it indicates productive infection (mRNA expression) in co-infected cats is disproportionately more inhibited than infection success (provirus); and *vice versa* for the opposite pattern. PERMANOVA analysis showed a significant difference (p < 0.01) between the FIV mRNA single-infected:co-infected ratio and the FIV provirus single-infected:co-infected ratio (Figure 4). For individual tissues the mRNA single-infected:co-infected ratio was significantly less than the provirus single-infected:co-infected ratio for liver, pre-scapular LN, ileum and colonic LN and there was a similar trend (p < 0.1) for spleen, mesenteric LN and tonsil (Figure 4 and Table 2). This result indicates that PLV/FIV co-infection disproportionately inhibited productive FIV infection in multiple tissues.

### 2.6. FIV mRNA Transcription Levels Greatly Exceed PLV in the Gastrointestinal Tract

**Figure 5.**
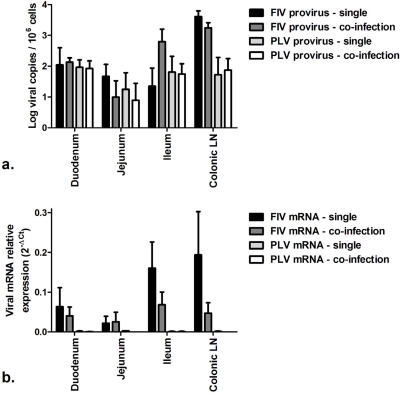
Comparison of FIV and PLV proviral load and viral mRNA load in gastrointestinal tissues and colonic LN. (**a**) FIV and PLV proviral loads in single-infected and co-infected cats. (**b**) FIV and PLV mRNA loads in single-infected and co-infected cats. Viral mRNA expression was measured relative to GAPDH expression.

Since FIV mRNA transcription (productive infection) appeared to be inhibited by co-infection with PLV, we evaluated whether PLV mRNA transcription might also be limited in domestic cat infection. Previously we found that PLV proviral load decreased during chronic infection to almost undetectable levels in PBMC and lymphoid tissue though provirus was detectable at low levels in gastrointestinal tissues [19]. In this study we confirmed that GI-associated tissues had among the highest PLV proviral loads at necropsy (Supplementary Table 1). We evaluated PLV mRNA load in tissues with proviral loads similar to FIV (GI tissues and colonic lymph node) and compared PLV and FIV proviral load and mRNA load. PLV proviral load from both single and co-infected cats was relatively similar to FIV proviral load in duodenum, jejunum and ileum; and approximately 10-fold lower than FIV proviral load in colonic lymph node (Figure 5a). In stark contrast to this result, PLV mRNA load was 60 to 1000-fold lower than FIV mRNA load in the same tissues (Figure 5b). Thus, PLV mRNA transcripts are present in extremely low abundance compared to FIV mRNA transcripts, suggesting that PLV productive infection is inhibited during chronic infection of domestic cats to a significantly greater extent than FIV infection in co-infected cats.

## 3. Experimental Section

### 3.1. Study Design and Tissue Collection

Experimental design and tissue sample collection were described in detail by TerWee *et al*. [26]. Briefly, 20 cats in a specific pathogen-free breeding colony at Colorado State University housed in an AAALAC-international accredited animal facility were randomly divided into 4 groups of 5 cats. At day 0, two groups were intravenously inoculated with PLV-1695 and two groups were sham inoculated. At day 28 post PLV infection, one of the PLV-infected groups and one of the sham groups were intravenously inoculated with FIV-C. This design resulted in 4 treatment groups (n = 5 cats/group): 1. PLV single infection, 2. PLV/FIV co-infection, 3. FIV single infection and 4. sham infection. At day 159 post PLV infection, cats were euthanized and tissue samples were collected and stored at −80 °C. All procedures were approved by the CSU Institutional Animal Care and Use Committee prior to initiation.

### 3.2. Genomic DNA Extraction

DNA was extracted from tissues using a method which combined bead-based disruption/homogenization using the FastPrep®-24 instrument (M.P. Biomedicals, Irvine, CA, USA) with the DNeasy Blood and Tissue Kit (QIAGEN, Valencia, CA, USA). Tissue samples of 40 mg were placed in Lysing Matrix A tubes (M.P. Biomedicals). Then 450 μL buffer ATL and 50 μL proteinase K (QIAGEN) were added to each tube. Tissues were homogenized by high speed bead disruption in the FastPrep®-24 Instrument for 40 seconds at a speed setting of 6.0. Resulting homogenate was centrifuged at 14,000 × g for 10 minutes and supernatant was transferred to a new microcentrifuge tube. The DNeasy Blood and Tissue Kit protocol was then followed to extract DNA. After extraction, DNA was eluted with 100 μL H_2_O and stored at −20 °C for use.

### 3.3. RNA Extraction and cDNA Synthesis

RNA was extracted from tissues using the FastRNA pro green kit (M.P. Biomedicals, Irvine, CA, USA) with FastPrep®-24 homogenizer (M.P. Biomedicals) following the manufacturer protocol. Briefly, 100 mg tissue was homogenized in RNA*pro*™ Solution and Lysing Matrix D using the FastPrep®-24 instrument for 40 seconds at a setting of 6.0. RNA was then purified according to the manufacturer’s instructions, resuspended in 100 μL RNase-free H2O and stored at −80 °C. Cellular RNA was then converted to cDNA using random primers and Superscript II (Invitrogen, Carlsbad, CA, USA) according to the manufacturer’s instructions.

### 3.4. Quantitation of Proviral Load, mRNA Viral Load and Cytokine Transcripts by Real-Time qPCR

PLV and FIV tissue DNA proviral loads were determined by qPCR using previously described assays targeting FIV-C gag [30] and PLV pol [31]. Due to high sequence heterogeneity between FIV and PLV, these primer-probe sets are specific for each virus and do not cross-amplify [26]. Briefly, 5 μL of tissue extracted DNA was quantitated by comparison to standard curves generated using plasmids containing the FIV-C gag or PLV pol. The number of cell equivalents for each DNA sample was determined as described by Terwee *et al*. [26].

FIV and PLV mRNA in tissues was quantified by qPCR using the previously described FIV-C gag assay [30] and PLV pol assay [31]. While these assays target two different lentiviral genes, the abundance of gag and pol-containing mRNAs during feline lentiviral infection is very similar [32], suggesting that these assays are reasonable for comparing viral mRNA levels of FIV and PLV. To allow accurate comparison between samples, viral mRNA expression was normalized to mRNA expression of glyceraldehyde-3-phosphate dehydrogenase (GAPDH) for each sample using the 2^−ΔCt^ method, in which ΔCt is the cycle at which threshold is reached for GAPDH subtracted from the cycle threshold for viral mRNA. Conversion of ΔCt to 2^−ΔCt^ produced a value which indicates the fold abundance of viral mRNA relative to that of GAPDH mRNA.

Cytokine mRNA expression was quantitated by qPCR as previously described [26,33]. Expression of IL-10, IL12p40 and IFNγ mRNA was quantitated relative to expression of GAPDH mRNA using the 2^−ΔCt^ method. All qPCR assays in the study were performed in triplicate and qPCR efficiencies were within the accepted range of 90–110%.

### 3.5. Statistical Analysis

Prior to PERMANOVA data were square root transformed to reduce the dominance of high proviral, FIV mRNA and cytokine levels, among individual tissues, on the analysis. PERMANOVAs were based on Bray-Curtis similarity matrices and graphically represented using non-metric multidimensional scaling (NMDS) plots. PERMDISP analyses were based on the same transformation and similarity matrix as PERMANOVA. GLM models were generally based on a normal data distribution and identity link function, except where a more optimal dispersion (poisson or gamma) or link function (log) could be identified, as determined by Akaike Information Criterion. All analyses described were undertaken using programs PRIMER [34] and R [35].

## 4. Discussion and Conclusions

Protection from FIV disease by infection with an avirulent, non-adapted lentivirus presents a model system for understanding factors which can protect mammalian hosts from virulent lentiviral diseases including HIV/AIDS. In this study we compared viral infection and innate immune response in multiple relevant tissue reservoirs for FIV/PLV co-infected and single virus infected cats. We determined PLV proviral load, FIV proviral load, FIV mRNA abundance and mRNA expression of IL-10, IL-12p40 and IFNγ for 12 different tissues from cats infected with FIV, PLV or both viruses. Using a multivariate approach we found that distribution of FIV provirus and IFNγ significantly differed between FIV single and PLV/FIV co-infected cats, while PLV proviral load, FIV mRNA, IL-10 and IL-12p40 did not. Co-infected cats had increased levels of FIV provirus and IFNγ in several tissues compared to single infected cats. Analysis of data dispersion demonstrated that variability in IFNγ and FIV mRNA was much more constrained in the co-infected cats than single infected cats, suggesting that these parameters are tightly regulated during co-infection. By comparing the single-infected:co-infected ratios of FIV mRNA to FIV provirus to control for the different scales used to measure these parameters, we found that production of FIV mRNA was inhibited in co-infected cats. Finally, we found that PLV mRNA production was highly restricted, suggesting that PLV itself is subject to inhibition of productive infection. Taken together, these results indicate that PLV infection impacts subsequent FIV infection by upregulation of IFNγ and alteration of establishment of tissue reservoirs. Both the magnitude and variability of productive FIV infection in multiple tissues was broadly inhibited in the face of prior PLV infection.

Lymphoid and gastrointestinal tissues represent important targets for lentiviral infection [36–38]. However, most studies of HIV infection in humans are limited to blood samples in which analysis of infection in circulating cells captures only a small fraction of the host infection. Animal models of virulent lentiviral disease provide a unique opportunity to examine infection and immunity in solid tissues which may differ from peripheral blood cells. We examined lentiviral co-infection and innate immune response in lymphoid tissues (bone marrow, thymus, spleen, mesenteric lymph node, pre-scapular lymph node, colonic lymph node and tonsil), gastrointestinal tissues (duodenum, jejunum, ileum and peyer’s patch) and liver. Overall, magnitude of viral infection and cytokine expression did not vary dramatically between lymphoid *versus* gastrointestinal tissues. Additionally, trends in co-infection *versus* single infection for FIV provirus and FIV mRNA were generally similar among different tissue types. The three tissues with significant differences in IFNγ expression between co-infection and single infection (thymus, spleen and pre-scapular LN) were all lymphoid tissue, but there was a similar near-significant trend in the duodenum. Thus, key effects attributed to PLV infection in this study were generally similar over a broad range of different tissue targets of infection. These data imply that possible impacts of PLV on cellular targets for FIV infection and cellular activation are likely mediated through cell subsets present in a wide range of tissue types.

We have previously detected increased IFNγ expression in PBMC of FIV/PLV co-infected cats (compared to FIV or PLV singly infected cats) at up to 59 days post-PLV infection [26]. In this study we found that IFNγ expression is also increased in multiple tissues of co-infected cats. In addition, we detected increased IFNγ at 159 days post-infection, which further extends the known duration of innate immune activation in co-infection and suggests that innate immune alterations induced by PLV persist into the chronic stage of FIV infection. Increased IFNγ expression occurs well beyond the end of apparent active PLV viral replication, as we found PLV to have extremely low mRNA abundance at this timepoint. The role of IFNγ in protection from feline and human AIDS is not clear [39]. However, Roberts *et al*. recently demonstrated a positive association between level of IFNγ in circulation during early infection and lower HIV viral load [40], providing a basis for the idea that IFNγ may promote protection from virulent lentiviruses. Thus, it is plausible that PLV-induced IFNγ may mediate activation of cellular defenses which inhibit productive FIV infection and hence reduce CD4+ T cell depletion with effects that last well beyond the initial infection. The reduced variability in IFNγ expression detected in co-infected cats may be indicative of constraints placed by PLV infection on IFNγ expression due to alteration of the cell types infected with FIV or activation state of IFNγ-expressing cells. Alternatively, it may be that consistent upregulation of IFNγ results in reduced sample-to-sample variability. While we chose to focus on IFNγ as an indicator of pro-inflammatory innate immunity, IFNα might also play a role in PLV/FIV host restriction and may be valuable to examine in future studies. The finding that tissue IL-10 and IL-12p40 mRNA expression did not differ significantly between infection groups is consistent with previous findings in PBMC [26], but is interesting since we have detected significantly increased IL-12p40 protein in the plasma of FIV and PLV/FIV infected cats (Britta Wood and Sue VandeWoude, in review), suggesting that mRNA and protein levels may not be tightly coupled for certain cytokines.

Previous characterization of FIV proviral load in PBMC of co-infected and single infected cats showed a slight trend toward greater levels of provirus in FIV single infected cats with a significant difference between the groups at one of eight timepoints studied [26]. Given these data and the reduced CD4+ T-cell depletion observed in co-infected cats, we were surprised to find that several tissues of co-infected cats had significantly higher FIV proviral load than single infected cats. This finding suggests that: (1) PBMC viral loads may not be indicative of tissue viral kinetics, and (2) the dynamics of FIV infection in the face of PLV infection change over time—a point illustrated by earlier studies of FIV quasispecies during co- and single infection [29]. The relevance and unpredictability of tissue *versus* peripheral viral pathogenesis highlights the importance of using animal models to study lentiviral infection at the tissue level.

The reasons for PLV-induced enhancement of FIV proviral load are unknown. PLV-induced alteration of cellular activation may have different consequences in different cell types, resulting in reduced or enhanced FIV infection or alterations in target cell availability and phenotype. While the overall levels of FIV provirus are equal or higher in co-infected cats than single infected cats, it still remains a distinct possibility that PLV infection alters the target cell population for FIV and that replication of FIV in an alternate cell type may ultimately lead to reduced CD4+ T-cell depletion. Furthermore, the finding that PLV infection does not reduce, and may enhance, successful FIV infection argues against the possibility of PLV-induced down-regulation of FIV entry receptor/co-receptor as a mechanism of protection since receptor down-regulation would be expected to result in a reduction in integrated FIV provirus. If PLV infection were to limit subsequent FIV infection to a cell type with a longer circulating half-life (*i.e.*, a cell with a memory phenotype) than the cell type preferred in a single infection, it is possible that proviral loads in co-infection could ultimately exceed those of single infection, as noted in this study.

Comparison of the single-infected:co-infected ratios of FIV provirus and FIV mRNA yielded a striking result: PLV co-infection resulted in strong inhibition of FIV mRNA transcription relative to FIV single infection in multiple infected tissues. CD4+ T-cell depletion during lentiviral infection requires productive viral infection and transcription regardless of whether depletion is ultimately driven by direct effects or chronic immune activation [41,42]. Thus, PLV-mediated intracellular inhibition of FIV transcription in multiple tissues may result in reduced CD4+ T-cell loss through a similar process. The exact mechanism underlying this observation is not clear. PLV might induce a state of latency in FIV-infected cells via cytokine regulation, or by redirecting FIV infection into alternate target cells which do not support active viral transcription. Alternatively, PLV-induced activation of cellular restriction factors might play a role in controlling productive FIV transcription. Our finding of extremely low PLV mRNA production suggests that PLV productive infection is restricted at an intracellular level. The finding of abundant G-to-A mutation in PLV genomes in infected cats [21] strongly implicates cellular cytidine deaminase-mediated restriction of PLV infection and suggests that PLV infection activates cellular restriction or cannot restrict basal intracellular restriction factors. The observation that PLV mRNA transcription in gastrointestinal tissues is severely restricted suggests that PLV genomes are defective and/or there is very effective host control of viral replication. While FIV genomes in infected cats did not have an excess of G-to-A mutations, individual hypermutated FIV genomes have been observed [21,29]. This suggests that FIV replication may also be affected, albeit to a lesser extent, by cellular cytidine deaminase restriction.

Our observation that FIV mRNA transcription is less muted during co-infection than PLV transcription, suggests that FIV is able to escape innate immune mechanisms that apparently control non-adapted PLV. The observation that PLV proviral loads are very low in most tissues, and that active viral replication is minimal in the face of continued elevated IFNγ transcription suggests that PLV infection works in *trans* to exert its effects on FIV co-infection viral and cytologic kinetics. The dual results that PLV mRNA transcription is highly inhibited and that FIV mRNA transcription is also inhibited in the context of PLV infection provide support for the idea that cellular restriction may mediate both effects.

PLV infection might limit cellular targets of infection for FIV, forcing FIV infection to take place in a cell type in which replication is more highly regulated by cellular restriction factors. Regardless of the mechanism of action, these data suggest PLV-induced inhibition of FIV transcription may impact the FIV disease state. It is further possible that during the FIV population ‘bottleneck’ previously described during co-infection [29], proviral genomes which have accumulated deleterious mutations through genetic drift survive the bottleneck, resulting in a lower transcription capacity following viral expansion.

Our data cumulatively suggest a model for PLV-induced protection from FIV disease in which PLV-induced innate immunity and/or intracellular defense result in inhibition of FIV transcription. This demonstrates that enhancement of early innate immune and cellular defense can alter the outcome of a virulent lentiviral infection. Future studies will be directed at further defining the basis for this protection by examining in greater detail how cellular subsets in blood and tissues, which may serve as anti-viral effectors or targets for infection, are altered by co-infection. Ultimately, we hope these studies will contribute to a more clear understanding of correlates of protection against lentiviruses and thereby direct therapies targeted at enhancing innate and cellular defense against HIV/AIDS.

## Supplementary Data



## Figures and Tables

**Table 1.** GLM analyses of differences between FIV single and PLV/FIV co-infected cats for FIV proviral load and IFNγ mRNA expression. Significant results in bold for all GLMs.

**Tissue**	**FIV Provirus**		**IFNγ**	
	***t*_1,8_**	***P***	***t*_1,8_**	***P***
Bone marrow	1.449	0.263	0.147	0.711
Thymus	2.105	0.185	7.315	**0.027**
Spleen	0.063	0.808	12.879	**0.007**
Liver	1.612	0.240	2.043	0.191
Pre-scapular LN *	19.305	**0.002**	7.843	**0.023**
Mesenteric LN *	5.355	**0.049**	0.264	0.621
Peyer’s patch	0.532	0.487	0.277	0.613
Duodenum	0.025	0.878	3.975	0.081
Jejunum	1.094	0.326	2.854	0.130
Ileum	6.539	**0.034**	1.735	0.224
Colonic LN *	2.185	0.178	0.262	0.623
Tonsil	0.001	0.981	1.960	0.199

* LN = lymph node.

**Table 2.** Inhibition of productive FIV infection in PLV/FIV co-infected cats (differences between FIV mRNA single:co-infected ratios and FIV provirus single:co-infected ratios). Significant GLM analysis results are in bold.

**Tissue**	**FIV mRNA and Provirus Single:Co Ratio**	
	***t*_1,8_**	***P***
Bone marrow	0.973	0.335
Thymus	0.850	0.400
Spleen	1.901	0.063
Liver	2.804	**0.007**
Pre-scapular LN *	2.076	**0.043**
Mesenteric LN *	1.909	0.062
Peyer’s patch	0.846	0.402
Duodenum	0.775	0.442
Jejunum	0.789	0.434
Ileum	2.854	**0.006**
Colonic LN *	2.116	**0.040**
Tonsil	1.722	0.092

* LN = lymph node.
