# Supplementary material for: Prior Virus Exposure Alters the Long-Term Landscape of Viral Replication during Feline Lentiviral Infection
Source: Viruses. 2011 Oct 13;3(10):1891–908. doi: 10.3390/v3101891 (PMC3205387; doi:10.3390/v3101891)
Supplement: Supplementary file 1 [file viruses-03-01891-s001.doc]

**Supplementary Table 1.** PLV proviral and FIV mRNA loads (Mean ± SE) among tissues for single and co-infected cats.

| **Tissue** | **Single** | | **Co-Infected** | |
| --- | --- | --- | --- | --- |
|  | **Mean** | **SE** | **Mean** | **SE** |
|  | PLV proviral copies/10^6^ cells | | | |
| Bone marrow | 0.00 | 0.00 | 10.70 | 6.65 |
| Thymus | 0.78 | 0.78 | 20.70 | 14.72 |
| Spleen | 230.78 | 149.45 | 94.75 | 65.37 |
| Liver | 7.14 | 5.72 | 16.62 | 7.51 |
| Pre-scapular LN * | 261.92 | 247.29 | 247.18 | 138.74 |
| Mesenteric LN * | 333.28 | 206.80 | 104.80 | 36.26 |
| Peyer's patch | 97.22 | 52.04 | 230.60 | 86.38 |
| Duodenum | 140.88 | 46.43 | 145.96 | 68.56 |
| Jejunum | 129.32 | 101.50 | 69.40 | 42.60 |
| Ileum | 276.60 | 158.38 | 120.10 | 51.02 |
| Colonic LN * | 505.64 | 428.83 | 268.81 | 190.49 |
| Tonsil | 10.02 | 4.92 | 11.51 | 9.64 |
|  | FIV mRNA relative expression (×10^−2^) | | | |
| Bone marrow | 61.34 | 30.65 | 10.29 | 2.76 |
| Thymus | 266.24 | 233.63 | 119.42 | 62.50 |
| Spleen | 122.70 | 95.62 | 17.28 | 8.89 |
| Liver | 2.31 | 1.30 | 0.07 | 0.02 |
| Pre-scapular LN * | 8.39 | 4.74 | 3.81 | 2.56 |
| Mesenteric LN * | 12.37 | 10.32 | 7.05 | 3.63 |
| Peyer's patch | 8.91 | 7.16 | 2.57 | 0.43 |
| Duodenum | 6.39 | 4.77 | 4.06 | 2.20 |
| Jejunum | 1.78 | 1.40 | 2.58 | 2.42 |
| Ileum | 16.06 | 6.58 | 6.85 | 3.21 |
| Colonic LN * | 19.40 | 10.87 | 4.71 | 2.62 |
| Tonsil | 14.46 | 10.28 | 1.92 | 0.77 |

^*^ LN = lymph node.

**Supplementary Table 2.** IL-10 and IL-12 mRNA expression (Mean ± SE) among tissues for FIV single and PLV/FIV co-infected cats.

| **Tissue** | **FIV Single** | | **PLV/FIV co-Infected** | |
| --- | --- | --- | --- | --- |
|  | **Mean** | **SE** | **Mean** | **SE** |
|  | IL-10 mRNA relative expression (×10^−3^) | | | |
| Bone marrow | 1.31 | 0.20 | 2.43 | 0.91 |
| Thymus | 11.43 | 5.80 | 17.09 | 5.42 |
| Spleen | 13.15 | 4.99 | 8.29 | 2.39 |
| Liver | 4.49 | 2.48 | 1.53 | 0.18 |
| Pre-scapular LN * | 7.94 | 2.83 | 16.42 | 4.25 |
| Mesenteric LN * | 3.13 | 0.91 | 5.52 | 0.71 |
| Peyer's patch | 24.72 | 18.73 | 9.98 | 1.06 |
| Duodenum | 6.04 | 4.81 | 6.60 | 2.72 |
| Jejunum | 1.00 | 0.66 | 5.44 | 3.94 |
| Ileum | 18.75 | 11.38 | 6.55 | 1.42 |
| Colonic LN * | 6.63 | 1.03 | 10.38 | 3.08 |
| Tonsil | 11.15 | 3.74 | 11.58 | 2.87 |
|  | IL-12 mRNA relative expression (×10^−3^) | | | |
| Bone marrow | 1.79 | 0.50 | 3.36 | 1.40 |
| Thymus | 189.05 | 86.39 | 47.40 | 16.98 |
| Spleen | 59.15 | 42.25 | 34.61 | 14.75 |
| Liver | 18.74 | 15.39 | 3.53 | 0.58 |
| Pre-scapular LN * | 48.46 | 18.47 | 127.10 | 49.32 |
| Mesenteric LN * | 24.33 | 4.37 | 23.34 | 4.23 |
| Peyer's patch | 15.21 | 6.63 | 29.53 | 9.10 |
| Duodenum | 65.48 | 55.57 | 17.29 | 5.00 |
| Jejunum | 6.65 | 2.50 | 16.00 | 7.38 |
| Ileum | 63.57 | 25.03 | 11.23 | 2.20 |
| Colonic LN * | 77.01 | 30.74 | 50.56 | 13.68 |
| Tonsil | 35.60 | 14.95 | 27.05 | 5.15 |

^*^ LN = lymph node.
